# Frequency of Classical Hand Signs in Cervical Spondylotic Myelopathy: A Prospective Cross-Sectional Observational Study

**DOI:** 10.7759/cureus.105506

**Published:** 2026-03-19

**Authors:** Ravi Ranjan Rai, Sandesh Subhash Agrawal, Bharat R Dave, Ajay Krishnan, Shivanand C Mayi, Mirant B Dave, Mikeson Panthackel, Mahesh Sagar

**Affiliations:** 1 Spine Surgery, Stavya Spine Hospital and Research Institute, Ahmedabad, IND; 2 Orthopaedics, Sri Devaraj Urs Academy of Higher Education and Research, Kolar, IND; 3 Orthopaedics and Traumatology, Shri Balaji Institute of Medical Sciences, Raipur, IND; 4 Spine Surgery, Shree Narayana Hospital, Raipur, IND; 5 Spine Surgery, Bhavnagar Institute Of Medical Science, Bhavnagar, IND

**Keywords:** cervical spondylotic myelopathy, finger escape sign, grip strength, hoffman’s sign, myelomalacia, prevalence, t2 hyperintensity

## Abstract

Background

Cervical spondylotic myelopathy (CSM) is a predominant cause of progressive neurological disability in ageing populations, frequently manifesting initially as subtle hand dysfunction before gait abnormalities. Early recognition is essential to prevent irreversible corticospinal damage. Although MRI remains the standard for anatomical assessment, bedside neurological evaluation combined with quantitative hand function testing may detect early functional compromise. This study aimed to determine the frequency of classical hand signs, quantify grip strength impairment, and correlate and evaluate their association with validated functional severity scales.

Methods and materials

This prospective cross-sectional observational study included 102 consecutive adults with clinically suspected and MRI-confirmed cervical spondylotic myelopathy treated at a tertiary spine center between August 2024 and January 2025. Demographic details, symptom profile, and neurological findings were recorded. Classical hand signs, including Hoffmann’s sign, finger escape sign, grip-and-release test, crossed radial reflex, and inverted radial reflex, were systematically assessed. Grip strength was objectively measured using a calibrated hand dynamometer. Functional severity was graded using the modified Japanese Orthopaedic Association (mJOA) score and Nurick's scale. Magnetic resonance imaging (MRI) was used to confirm the diagnosis. Categorical variables were summarized using frequencies and percentages. Associations between clinical hand signs and functional severity scores were assessed using Pearson correlation analysis, with statistical significance set at p < 0.05.

Results

A total of 102 patients were included, predominantly elderly, with 79/102 (77.5%) aged ≥60 years and a male preponderance of 78/102 (76.5%). Gait imbalance was the most common presenting symptom, reported in 75/102 (73.5%), followed by impaired hand dexterity in 58/102 (56.9%). At least one classical hand sign was present in 73/102 (71.6%) patients. Hoffmann’s sign was positive in 49/102 (48.0%), the finger escape sign in 44/102 (43.1%), and the grip-and-release test in 17/102 (16.7%). Most patients demonstrated moderate functional impairment, with the highest frequency of mJOA scores between 10-12 (42/102, 41.2%) and Nurick's grades 3-4 (56/102, 54.9%). Hand signs showed significant correlation with disease severity, demonstrating a negative correlation with mJOA score (r = −0.40, p < 0.001) and a positive correlation with Nurick's grade (r = 0.433, p < 0.001).

Conclusions

Classical hand signs and grip impairment were frequently observed in cervical spondylotic myelopathy and showed a significant association with functional severity scores (mJOA and Nurick). Structured bedside neurological examination may serve as a practical adjunct to imaging in assessing disease severity.

## Introduction

Cervical spondylotic myelopathy (CSM) results from degenerative changes in the cervical spine leading to spinal cord compression. The hallmark symptoms include loss of hand dexterity, weakness, numbness, and impaired fine motor skills - such as difficulty with buttoning, writing, or typing. These hand dysfunctions are often among the earliest and most disabling features of CSM, sometimes preceding gait disturbances or other neurological deficits [1,2]. These signs, especially when combined, significantly increase the likelihood of CSM. The combination of multiple hand signs (e.g., Hoffmann, Babinski, finger escape) improves diagnostic accuracy and should prompt further investigation with imaging [3]. The Trömner sign and hyperreflexia are highly sensitive for screening, while the Babinski sign, clonus, and inverted supinator sign are specific for confirming CSM [4]. The finger escape sign and grip-and-release test are beneficial for early detection and quantification of hand dysfunction [5]. Hand signs are central to the clinical diagnosis of CSM, providing sensitive and specific indicators of spinal cord dysfunction. However, no single sign is perfectly sensitive or specific, and some patients may lack classic findings despite significant cord compression [6]. Recognising these signs enables timely intervention, improving patient outcomes and reducing the risk of permanent disability [5,6]. MRI findings, such as cord compression ratio and signal changes, correlate with the severity of hand signs and symptoms, although not all clinical signs predict the exact level or degree of compression. The primary objective of this study was to determine the frequency of classical neurological hand signs in patients with cervical spondylotic myelopathy and to evaluate their association with validated functional severity scales (modified Japanese Orthopaedic Association and Nurick grade).

## Materials and methods

This prospective cross-sectional observational study was conducted at Stavya Spine Hospital and Research Institute, Ahmedabad, India, from August 1, 2024, to January 1, 2025. Institutional Ethics Committee approval was obtained (Protocol No.: SSHRI/CS/NS/Handsign/RR/63/07.24), and written informed consent was obtained from all participants. The objectives of this study were to determine the frequency of classical neurological hand signs in patients with cervical spondylotic myelopathy, to quantify grip strength impairment using dynamometric assessment, and to evaluate the association between these clinical findings and functional severity as measured by the modified Japanese Orthopaedic Association (mJOA) score and Nurick grade.

Participants

A total of 102 consecutive adult patients (≥18 years) with clinically suspected and MRI-confirmed cervical spondylotic myelopathy were enrolled. Inclusion criteria were adults aged ≥18 years with clinically suspected cervical myelopathy and MRI-confirmed cervical spinal cord compression. Exclusion criteria included prior cervical spine surgery, acute traumatic spinal cord injury, inflammatory or infectious spinal pathology, peripheral neuropathy, or other neurological disorders affecting hand function.

Clinical assessment

All patients underwent a standardized neurological examination performed by a spine surgeon. Classical hand signs, including Hoffmann’s sign, finger escape sign, grip-and-release test, inverted radial reflex, and crossed radial reflex, were assessed using established clinical techniques. Grip strength was measured using a calibrated hand dynamometer, and the best of three attempts was recorded for analysis. Functional severity was graded using the mJOA score and Nurick grade. A positive Hoffmann’s sign was defined as reflex thumb flexion following flicking of the distal phalanx of the middle finger. The finger escape sign was considered positive when spontaneous abduction and flexion of the ulnar digits occurred upon sustained finger extension. Grip-and-release test abnormality was defined as fewer than 20 complete cycles within 10 seconds.

Imaging assessment

All patients underwent cervical spine magnetic resonance imaging (MRI) using a 1.5-Tesla system. The imaging protocol included sagittal T1-weighted and T2-weighted sequences, along with axial T2-weighted images at the levels of interest. Slice thickness ranged from 3-4 mm. Images were systematically evaluated for the level and degree of spinal cord compression, canal stenosis, and the presence of intramedullary T2 hyperintensity suggestive of myelomalacia. MRI findings were used to confirm the diagnosis and characterize structural severity. Functional severity was graded using the mJOA score [7] and Nurick's grade [8].

Sample size estimation

A priori sample size estimation was performed for correlation analysis between neurological hand signs and functional severity scores (mJOA and Nurick). Assuming a moderate correlation coefficient (r = 0.35), with a two-tailed alpha of 0.05 and 80% statistical power, the minimum required sample size was calculated to be 90 participants. The inclusion of 102 consecutive eligible patients, therefore, provided adequate statistical power to detect clinically meaningful associations.

Statistical analysis

Statistical analyses were performed using standard statistical software. Continuous variables were expressed as mean ± standard deviation, and categorical variables were presented as frequencies and percentages. The association between neurological hand signs and functional severity scores (mJOA and Nurick) was evaluated using Pearson correlation analysis. A two-tailed p-value < 0.05 was considered statistically significant.

## Results

Demographic characteristics

**Figure 1:**
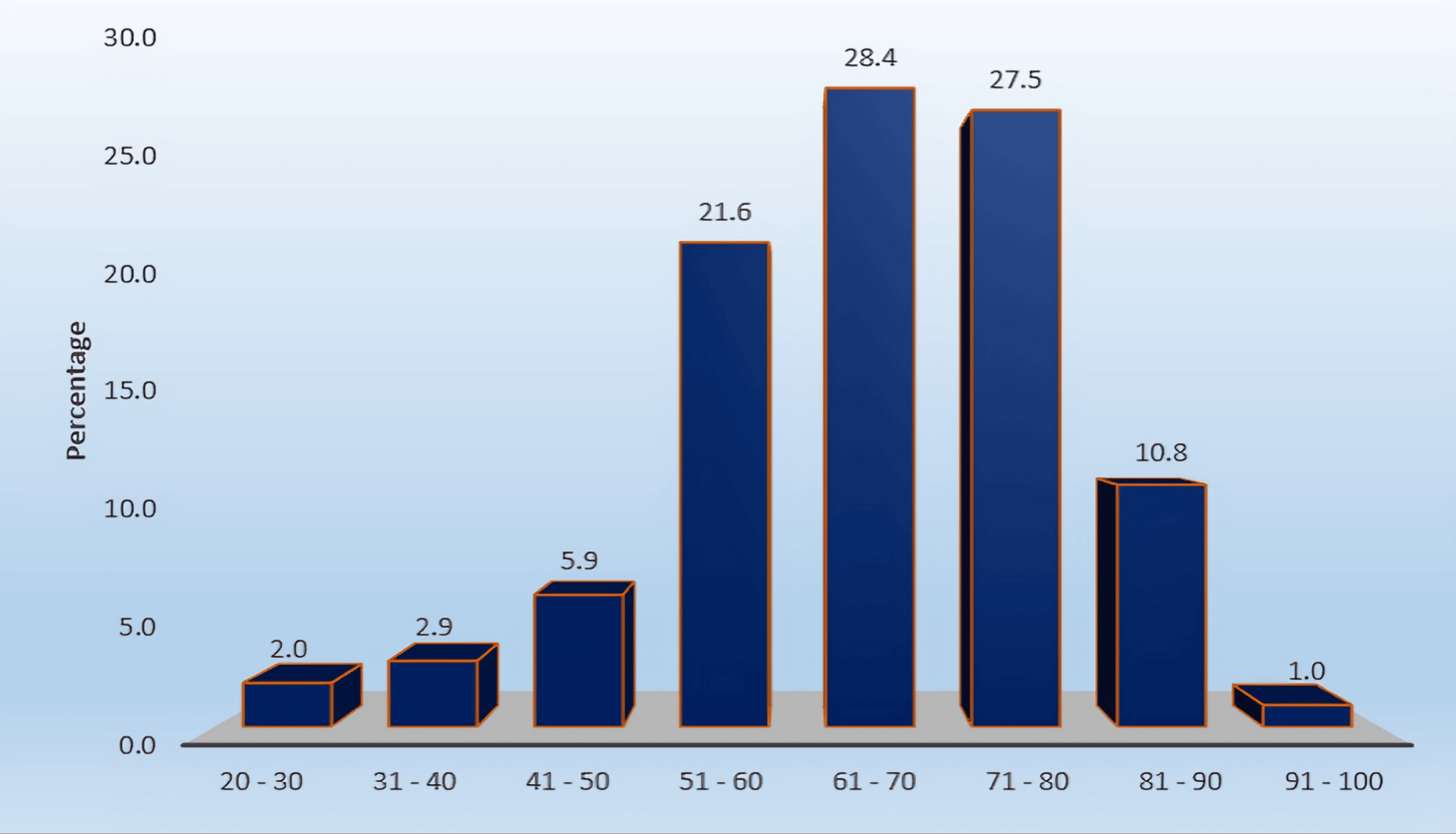
Age-wise distribution of study participants (n = 102) Distribution of patients across age categories.

A total of 102 consecutive patients fulfilling the clinical and radiological criteria for cervical spondylotic myelopathy were included in the final analysis. The study population predominantly comprised older adults, with the majority clustered in the sixth to eighth decades of life. Patients aged 61-70 years constituted 29/102 (28.4%), closely followed by those aged 71-80 years (28/102, 27.5%). The 51-60 year age group also represented a substantial proportion of the cohort, further supporting the predominance of middle-aged and elderly individuals in the study population, as illustrated in Figure 1. Overall, 90/102 patients (88.2%) were older than 50 years, indicating a strong age-related distribution of cervical spondylotic myelopathy. Patients older than 80 years accounted for 12/102 (11.8%), while individuals younger than 40 years constituted 5/102 (4.9%) of the cohort. A marked sex disparity was observed. Men constituted 78/102 (76.5%), whereas women accounted for 24/102 (23.5%), yielding a male-to-female ratio of 3.25:1, as illustrated in Table 1.

**Table 1:** Sex distribution of study participants (n = 102) Majority of the cohort comprised male patients

Sex	Frequency	Percent
F	24	23.5
M	78	76.5
Total	102	100.0

Clinical presentation

With respect to presenting complaints, gait disturbance was the most frequently reported symptom, present in 75/102 patients (73.5%). Reported difficulties included imbalance, unsteady ambulation, and reduced walking endurance. Hand dysfunction, characterized by impaired dexterity, clumsiness, and difficulty performing fine motor tasks, was noted in 58/102 patients (56.9%). These symptoms frequently coexisted, and several patients presented with concurrent gait and hand involvement at the time of evaluation.

Distribution of neurological hand signs

Classical upper motor neuron signs affecting hand function were commonly observed during neurological examination. Overall, 73/102 patients (71.6%) demonstrated at least one positive pathological hand sign, while 29/102 (28.4%) had no demonstrable hand signs. Hoffmann’s reflex was the most frequently elicited sign, detected in 49/102 patients (48.0%). Among these, bilateral positivity was observed in 42/102 patients (41.2%), while unilateral positivity was noted in 7/102 patients (6.8%), as summarized in Table 2. The finger escape sign was present in 44/102 patients (43.1%). In contrast, the grip-and-release test showed abnormal performance in 17/102 patients (16.7%), making it the least frequently positive among the evaluated signs, as described in Tables 2-5.

**Table 2:** Distribution of Hoffmann’s sign (n = 102) Presence of unilateral or bilateral Hoffmann’s reflex.

Hoffmann's sign	Frequency	Percent
Bilateral positive	42	41.2
Unilateral positive	7	6.8
Negative	53	52.0
Total	102	100.0

**Table 3:** Distribution of finger escape sign (n = 102) Positive sign indicates progressive finger abduction due to intrinsic muscle weakness.

Finger escape sign	Frequency	Percent
No	58	56.9
Yes	44	43.1
Total	102	100.0

**Table 4:** Distribution of grip-and-release test results (n = 102) Abnormal test denotes reduced speed and coordination of repetitive hand movements.

Grip-and-release test	Frequency	Percent
Bilateral positive	12	11.8
Unilateral positive	5	4.9
Negative	85	83.3
Total	102	100.0

**Table 5:** Overall prevalence of any pathological hand sign (n = 102) Positive indicates presence of at least one abnormal neurological hand sign.

Hand sign	Frequency	Percent
Positive	73	71.6
Negative	29	28.4
Total	102	100

Distribution and overlap of upper motor neuron clinical signs

Overlap among upper motor neuron signs was observed in the 73 patients demonstrating at least one pathological hand sign. Hoffmann’s sign was present in 49 patients, finger escape sign in 44 patients, and grip-and-release test positivity in 17 patients. Among these, isolated Hoffmann’s sign was observed in 22 patients, isolated finger escape sign in 17 patients, and isolated grip-and-release impairment in 2 patients. Combined positivity was also observed, with Hoffmann’s sign and finger escape sign occurring together in 17 patients, Hoffmann’s sign with grip-and-release impairment in five patients, and finger escape sign with grip-and-release impairment in 5 patients. All three signs were simultaneously present in five patients. These findings demonstrate that Hoffmann’s sign was the most frequently observed clinical indicator and showed the greatest overlap with other upper motor neuron signs.

Functional severity assessment

Functional evaluation demonstrated a broad spectrum of neurological impairment. The mJOA scores ranged from 4 to 16. Most patients were concentrated between scores of 10 and 12 (42/102, 41.2%), corresponding to mild-to-moderate disability, while fewer patients exhibited severe impairment (scores ≤8). Similarly, the Nurick grading system demonstrated that grade 3 was the most common category (33/102, 32.4%), followed by grades 2 and 4. Severe ambulatory limitation (grade 5) was observed in approximately one-fifth of the cohort (21/102, 20.3%), as shown in Tables 6-7.

**Table 6:** Distribution of mJOA scores (n = 102) Lower scores represent greater functional impairment. mJOA: modified Japanese Orthopaedic Association

mJOA score	Frequency	Percent
4	3	2.9
5	3	2.9
6	7	6.9
7	8	7.8
8	5	4.9
9	11	10.8
10	11	10.8
11	15	14.7
12	16	15.7
13	7	6.9
14	9	8.8
15	6	5.9
16	1	1.0
Total	102	100.0

**Table 7:** Distribution of Nurick's grades (n = 102)

Nurick’s grade	Frequency	Percent
1	2	2.0
2	23	22.5
3	33	32.4
4	23	22.5
5	21	20.3
Total	102	100.0

Correlation analysis

**Figure 2:**
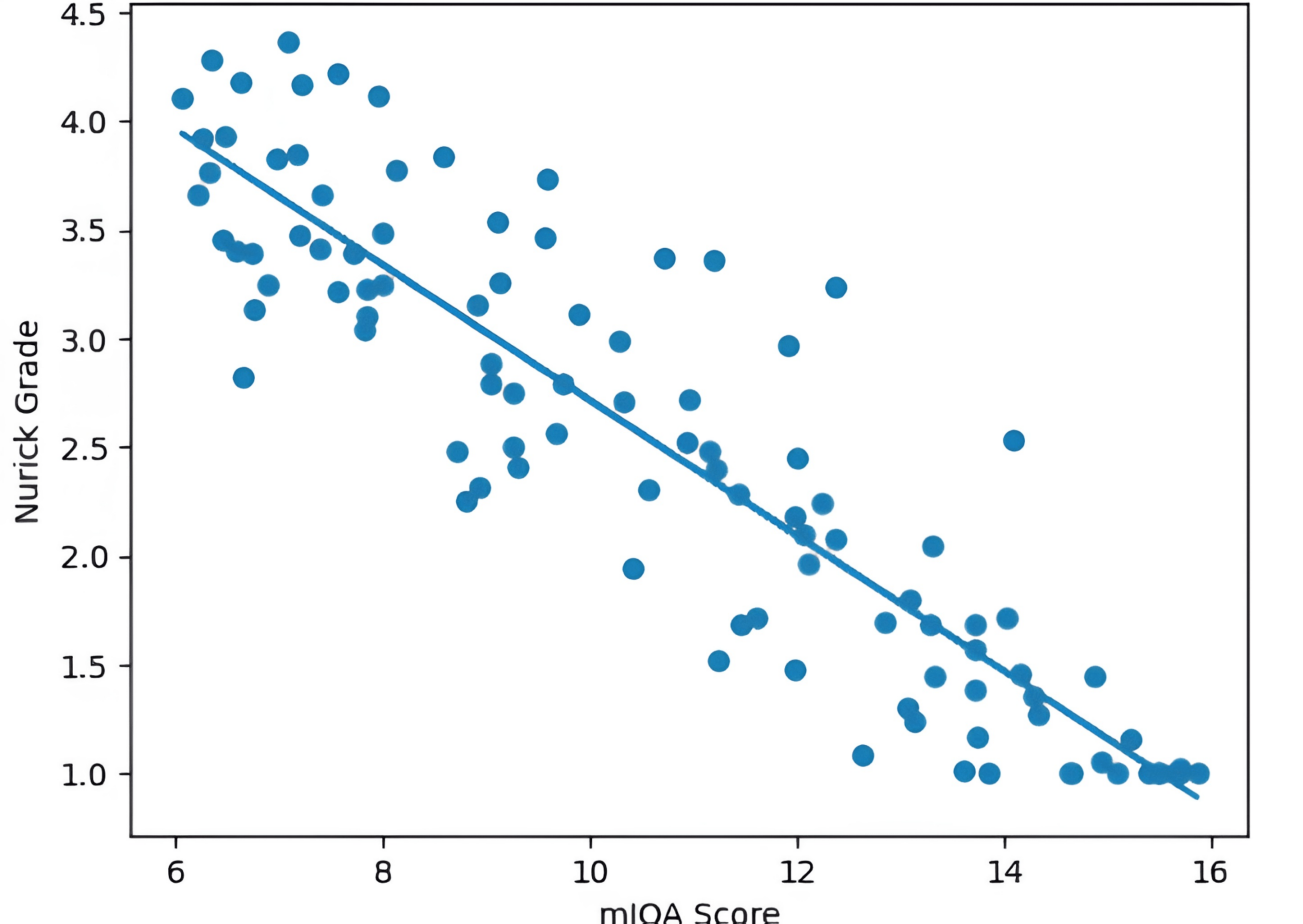
Scatter plot demonstrating the inverse correlation between mJOA score and Nurick grade in patients with cervical spondylotic myelopathy (r = −0.726, p < 0.001). mJOA: modified Japanese Orthopaedic Association

Correlation analysis demonstrated statistically significant associations between neurological hand signs and functional severity indices. The presence of pathological hand signs showed a moderate negative correlation with mJOA scores (indicating worse neurological status with lower scores) and a moderate positive correlation with Nurick grade (reflecting greater gait disability with higher grades). There was a significant inverse correlation between mJOA score and Nurick grade (r = −0.726, p < 0.001), as illustrated in Figure 2.

## Discussion

Degenerative cervical myelopathy (DCM) is the most common cause of non-traumatic spinal cord dysfunction in the elderly and results from chronic mechanical compression and ischemic injury to the cervical cord [1,2]. The present cross-sectional study involving 102 patients with cervical spondylotic myelopathy demonstrated a high frequency of classical hand signs and a significant correlation between bedside neurological findings and validated functional severity scales. These findings highlight the continued clinical relevance of structured bedside neurological examination as a complementary tool alongside imaging for assessing disease severity. Unlike previous research that focuses on radiological findings, this approach highlights the practical applicability and accessibility of bedside examinations in predicting disease severity.

The age distribution in our series showed a clear predominance of elderly individuals, with the majority of patients clustered in the sixth to eighth decades of life. This finding is consistent with the degenerative pathogenesis of DCM, where progressive disc degeneration, osteophyte formation, facet hypertrophy, and ligamentum flavum thickening cumulatively narrow the canal and compress the cord [3,4]. Similar peak incidence in older populations has been reported in large epidemiological studies and systematic reviews [5,6]. The male predominance in our cohort (76.5%) mirrors previous reports and may be due to greater occupational mechanical stress and higher exposure to risk factors for cervical degeneration [5,7].

Gait imbalance was the most common presenting symptom, followed by impaired hand dexterity. This pattern reflects early involvement of the corticospinal tracts and posterior columns, leading to spastic gait disturbance and loss of proprioception [6,8]. Hand clumsiness and reduced fine motor coordination are classical features of “myelopathy hand,” first described by Ono et al., characterized by difficulty in rapid grip-and-release and intrinsic muscle weakness [4]. Similar clinical presentations have been consistently documented in natural history studies of cervical spondylotic myelopathy [9,10,11].

Upper motor neuron signs were frequently observed in our patients. Hoffmann’s sign was positive in nearly half of the cohort, while the finger escape sign was present in 43.1% of cases. These signs are well-recognized indicators of corticospinal tract dysfunction and improve clinical detection of myelopathy when used together [9,12]. MRI findings, such as cord compression ratio and intramedullary signal intensity changes, have been reported to correlate with the severity of neurological deficits and functional impairment, although imaging findings do not always precisely predict the clinical level or severity of myelopathy [10,13]. The grip-and-release test showed lower positivity, likely because it becomes abnormal mainly in more advanced motor impairment. Overall, the presence of at least one pathological hand sign was observed in 71.6% of patients, indicating that subtle intrinsic hand dysfunction may precede more overt pyramidal signs. This supports earlier descriptions that hand dysfunction is one of the earliest and most sensitive clinical manifestations of DCM [9,13]. Assessment revealed a broad spectrum of neurological impairment. Most patients had moderate scores on the mJOA scale, indicating established yet potentially reversible dysfunction. The mJOA score is widely accepted for quantifying motor, sensory, and sphincter deficits in DCM and has demonstrated strong reliability and responsiveness [1,13]. Similarly, more than half of our patients were categorized as Nurick grades 3-5, reflecting significant gait disability. The Nurick grading system remains particularly useful for assessing ambulatory capacity and disease progression [11].

Correlation analysis further validated the clinical significance of these findings. The moderate negative correlation between hand signs and mJOA score and the positive correlation with Nurick grade indicate that increasing hand dysfunction parallels worsening neurological status and gait disability. The observed correlations suggest that hand sign positivity is associated with greater functional impairment. The strong negative correlation between mJOA and Nurick scores confirms the internal consistency and complementary nature of these two validated scales, as reported in previous outcome studies [12,13]. These results suggest that simple bedside clinical signs can reliably reflect functional severity and may serve as a useful adjunct clinical indicator, though diagnostic sensitivity and specificity were not evaluated in this study.

The continued importance of clinical examination is especially relevant in resource-limited settings where advanced imaging may not be accessible. Several studies have shown that delayed diagnosis contributes to irreversible spinal cord injury and poorer postoperative recovery [14]. Early recognition of myelopathic signs facilitates timely surgical referral, and multiple investigations have demonstrated better neurological recovery when decompression is performed before advanced disability develops [15-17]. Therefore, incorporating easily reproducible signs such as Hoffmann's reflex, finger escape sign, and especially the hand sign may enhance early screening and prognostication.

Pathophysiologically, chronic compression leads to demyelination, neuronal loss, microvascular compromise, and gliosis within the spinal cord, explaining the progressive and sometimes irreversible nature of neurological deficits [15]. Radiological and histopathological studies have shown that longer symptom duration is associated with poorer functional recovery, underscoring the need for early clinical detection [16]. Early demyelination and axonal injury may contribute to progressive neurological deterioration, underscoring the importance of early diagnosis and timely intervention to prevent irreversible neurological damage. Thus, bedside neurological markers that correlate with functional scales, as shown in this study, may have important implications for both diagnosis and timing of intervention.

Certain limitations should be acknowledged. The single-centre cross-sectional design may limit generalizability, and longitudinal outcomes or postoperative recovery were not evaluated. Imaging and electrophysiological correlations were not included, which could have strengthened diagnostic accuracy. Nevertheless, the adequate sample size and systematic assessment of multiple clinical signs provide a comprehensive evaluation of the relationship between bedside findings and functional impairment. Additionally, the cross-sectional design precludes assessment of causal relationships between hand signs and disease progression. Therefore, the independent predictive value of individual hand signs could not be determined. The absence of a non-myelopathic control group precludes assessment of the diagnostic accuracy and comparative prevalence of hand signs in asymptomatic or radiculopathy populations.

Overall, this study reinforces the importance of careful neurological examination in assessing DCM. The high prevalence of hand sign positivity and its significant correlation with validated severity scales suggest that this simple test may serve as a useful adjunct clinical indicator, though diagnostic sensitivity and specificity were not evaluated in this study. Integrating these clinical parameters into routine evaluation may aid early diagnosis, guide treatment decisions, and improve functional outcomes. Future larger-scale multicenter studies may further clarify the relationship between clinical hand dysfunction, radiological severity, and disease progression, thereby refining early diagnostic strategies.

## Conclusions

Hand dysfunction represents an early and highly prevalent manifestation of cervical spondylotic myelopathy. Classical bedside neurological signs, including Hoffmann’s sign, the finger escape sign, and particularly the composite hand sign, were frequently observed and demonstrated significant correlation with validated functional severity scales such as the modified JOA score and Nurick grade. Quantitative impairment of grip strength and dexterity reliably reflected corticospinal tract involvement and paralleled increasing neurological disability. These findings highlight that structured clinical examination remains a sensitive, practical, and cost-effective tool for early detection and severity assessment of myelopathy, often complementing MRI findings. Incorporating systematic evaluation of hand signs into routine assessment may facilitate earlier diagnosis, improve risk stratification, and support timely surgical intervention, thereby potentially preventing irreversible neurological deterioration and optimizing functional outcomes.
